# Cartilaginous Metabolomics Reveals the Biochemical-Niche Fate Control of Bone Marrow-Derived Stem Cells

**DOI:** 10.3390/cells11192951

**Published:** 2022-09-21

**Authors:** Haining Peng, Yi Zhang, Zhongkai Ren, Ziran Wei, Renjie Chen, Yingze Zhang, Xiaohong Huang, Tengbo Yu

**Affiliations:** 1 Department of Sports Medicine, The Affiliated Hospital of Qingdao University, Qingdao 266000, China; 2 Institute of Sports Medicine and Rehabilitation, Qingdao University, Qingdao 266000, China; 3 Shandong Institute of Traumatic Orthopedics, Medical Research Center, The Affiliated Hospital of Qingdao University, Qingdao 266590, China; 4 Department of Orthopedics, The Third Hospital of Hebei Medical University, Shijiazhuang 050051, China; 5 Department of Orthopedics, The Affiliated Hospital of Qingdao University, Qingdao 266000, China

**Keywords:** metabolomics, endogenous biomolecules, bone marrow-derived stem cells, chondrogenic differentiation, regeneration, cartilage

## Abstract

Joint disorders have become a global health issue with the growth of the aging population. Screening small active molecules targeting chondrogenic differentiation of bone marrow-derived stem cells (BMSCs) is of urgency. In this study, microfracture was employed to create a regenerative niche in rabbits (n = 9). Cartilage samples were collected four weeks post-surgery. Microfracture-caused morphological (n = 3) and metabolic (n = 6) changes were detected. Non-targeted metabolomic analysis revealed that there were 96 differentially expressed metabolites (DEMs) enriched in 70 pathways involved in anti-inflammation, lipid metabolism, signaling transduction, etc. Among the metabolites, docosapentaenoic acid 22n-3 (DPA) and ursodeoxycholic acid (UDCA) functionally facilitated cartilage defect healing, i.e., increasing the vitality and adaptation of the BMSCs, chondrogenic differentiation, and chondrocyte functionality. Our findings firstly reveal the differences in metabolomic activities between the normal and regenerated cartilages and provide a list of endogenous biomolecules potentially involved in the biochemical-niche fate control for chondrogenic differentiation of BMSCs. Ultimately, the biomolecules may serve as anti-aging supplements for chondrocyte renewal or as drug candidates for cartilage regenerative medicine.

## 1. Introduction

Age-related changes in the chondrocyte renewal in the articular cartilage predispose individuals to osteoarthritis [1,2]. Regenerative medicine aims to restore tissue functionality by harnessing the differentiation potential of stem cells in tissue replacement therapies [3]. Microfracture (MF) is a commonly used surgical method to stimulate cartilage regeneration, involving drilling on the subchondral bone to access the resident bone marrow-derived stem cells (BMSCs) [4] and creating a biochemical niche to induce chondrogenesis and sustain chondrocyte functionality [5]. However, the outcome of MF is correlated with age; i.e., greater improvement is only shown in young patients [6]. This may be due to BMSC aging, failure in chondrogenic differentiation, and dysfunction of the resident chondrocytes in old people [7,8]. Thus, the fate of the activated BMSCs plays a determinative role in the modeling and remodeling of cartilage, which are affected by both intrinsic and extrinsic factors.

The biochemical niche which is created by a variety of small molecules (metabolites) filling in the defect site in MF controls the fate of the activated resident BMSCs [9,10]. For example, omega-3 polyunsaturated fatty acids (ω-3 PUFAs), including docosapentaenoic acid 22n-3 (DPA) [11], maintain the self-renewal of embryonic stem cells [12]. The amino acids, such as tryptophan, modulate the senescence of mesenchymal stromal/stem cells (MSCs) by regulating mitochondrial integrity and function [13]. Yet, the excess reactive oxygen species (ROS), oxidative markers, impair stem cell self-renewal capacity [1,14,15]; subsequentially, the ROS-induced DNA damage causes replicative senescence in MSCs [16]. The excess ROS and related oxidative stress are correlated with the progression of osteoarthritis, characterized by the changes in the hyaline cartilage markers, i.e., an increased catabolism of proteoglycan aggrecan (ACAN) and a loss of type II collagen (COL2A1) [17]. Moreover, dietary intake of ω-3 PUFAs attenuates osteoarthritis-associated cartilage degradation [18]. Hence, we hypothesize that the metabolic cues in the regenerative niche contribute to BMSC activity and rebalance a hostile joint environment, which is of paramount importance for the fate control of BMSCs in chondrogenesis.

The aim of this study was to discover biomolecules with the potential to serve as anti-aging supplements for chondrocyte renewal or as drug candidates for cartilage regenerative medicine. In this study, MF was employed in healthy rabbits to create a regenerative niche for BMSCs, which maximizes the detection of the key components in cartilage regeneration and excludes the background noise in deteriorative joints. Non-targeted metabolomics was performed to determine the metabolic differences between the normal (NOR) and regenerated (REG) cartilages. A list of cartilage regeneration-related endogenous small molecules, i.e., the differentially expressed metabolites (DEMs), were identified. Two of the metabolites, DPA and ursodeoxycholic acid (UDCA), were picked to determine their specific effects on cartilage regeneration, i.e., BMSC vitality, chondrogenic differentiation, and chondrocyte functionality, based on the chondrocyte-protective role of ω-3 PUFAs [19] and the chondrocyte-differentiation-facilitating role of cholesterol (the substrate of UDCA) [20].

## 2. Materials and Methods

### 2.1. Rabbit Microfracture Model

**Figure 1:**
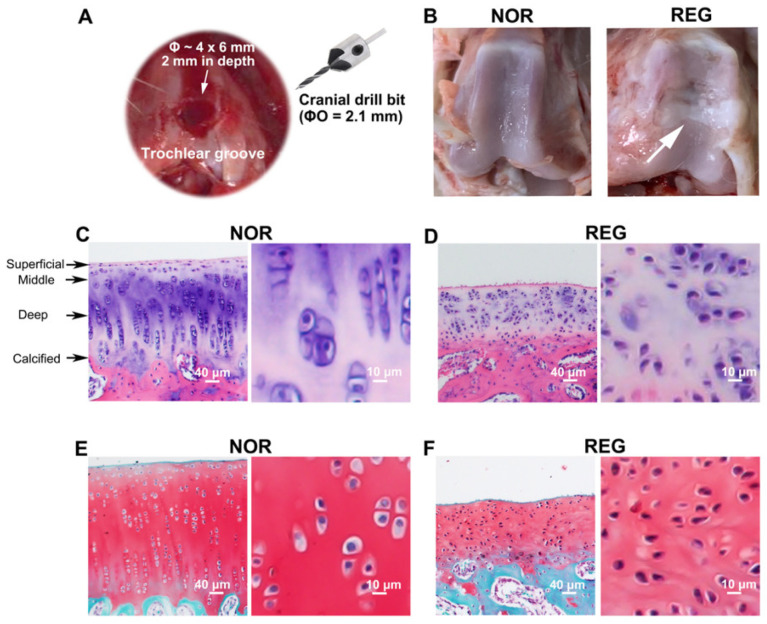
The histological profiles of normal and regenerated cartilages in rabbits. (**A**). The microfracture (MF) surgery is conducted on the trochlear groove of the left distal femur. An articular cartilage defect (Ø ~ 4 × 6 mm, 2 mm in depth) has been created on the trochlear groove of the left distal femur with a sterilized cranial drill bit (ØO = 2.1 mm). (**B**). The photographs of the trochlear grooves. Compared to the normal (NOR) cartilage, the regenerated (REG) cartilage loses its transparent color and smooth and glistening appearance, replaced with a rough surface with fissures. (**C**,**D**) Examples of the H&E staining. Compared to the NOR cartilage, the REG cartilage loses the highly organized structure composed of four zones, i.e., the superficial, middle, deep, and calcified zones. (**E**,**F**) Example of the Safranin-O/Fast Green staining. Compared to the NOR cartilage, the reddish-stained ACAN content in the REG cartilage was lower. The scale bars represent 40 μm and 10 μm.

The animal study was approved by the Ethics Committee of Experimental Animals of the Affiliated Hospital of Qingdao University (No. AHQU-MAL20210419). A self-controlled design was used in this study. Male New Zealand white rabbits (10–12 weeks in age, 2.0–2.5 kg in weight, n = 9) were anesthetized through ear vein administration of 3% *w*/*v* pentobarbital sodium at a dosage of 1.0 mg/kg. After sterilization, a 2–3 cm anteromedial parapatellar incision was made on the left knee, and the patella was everted. An articular cartilage defect (Ø ~ 4 × 6 mm, 2 mm in depth) was created on the trochlear groove of the left distal femur with a sterilized cranial drill bit (ØO = 2.1 mm, RWD, Shenzhen, China) (Figure 1A). Afterward, the debris was removed and hemostasis was achieved. All rabbits were treated with gentamycin for three days after the surgery.

### 2.2. Tissue Collection

The rabbits were sacrificed by CO_2_ asphyxiation, and the paired cartilage samples from both the left and right distal femurs (n = 9) were collected based on the reported protocol [19]. For histochemical analysis (n = 3), three pairs of trochlear grooves were kept in 4% paraformaldehyde in phosphate-buffered saline (PBS) for 24 h and then decalcified in EDTA (14% *w*/*v*, pH 7.4) for 21 days at 4 °C until measurement. For metabolomic analysis (n = 6), six pairs of cartilage tissues were washed with PBS and then stored at −80 °C until analysis.

### 2.3. Histochemistry

The decalcified tissues were embedded in paraffin, and then sagittal sections were cut at 3 μm. Hematoxylin–eosin (H&E) staining was conducted to determine the cellular regularity of cartilage using a commercial kit (G1120, Solarbio, Beijing, China) according to the published procedure [20]. A commercial kit (G1371, Solarbio) was used for the Safranin-O/Fast Green staining, in which the depth of the reddish color is correlated with the content of ACAN [21], by following the company’s instructions. All the slices were dehydrated, transparentized, and then mounted with neutral resin for microscopic observation (Leica, Herlev, Denmark). Images were representative results of three biological repeats.

### 2.4. Metabolomics

#### 2.4.1. Sample Preparation for Liquid Chromatography–Mass Spectrometry

L-2-Chlorophenylalanine (20 µL, 0.3 mg/mL in methanol) was used as the internal standard. The cartilage sample (30 mg) was mixed with ice-cooled methanol (400 µL, 80%), precooled for 2 min at −20 °C, and then ground at 60 Hz for 2 min. After ultrasonic extraction, the solution was centrifuged at 13,000 rpm for 10 min at 4 °C. Then, 300 µL of the supernatant was evaporated and re-dissolved with 200 µL methanol (20%). The mixture was incubated for 2 h at −20 °C and then centrifuged at 11,400× *g* (TGL-16MS, Shanghai Lu Xiangyi Centrifuge Instrument Co., Ltd., Shanghai, China) for 10 min at 4 °C. Afterward, 150 µL of the liquid supernatant from each tube was collected, filtered by an organic phase pinhole filter (0.22 μm), and then stored at −80 °C until analysis.

#### 2.4.2. Liquid Chromatography–Mass Spectrometry

Liquid chromatography–mass spectrometry (LC-MS) analysis was conducted by following a published protocol [22]. Briefly, the LC was performed using an ACQUITY UPLC HSS T3 1.8-micron column at 45 °C. The mobile phase contained water and acetonitrile with 0.1% formic acid; gradient elution was conducted at a flow rate of 0.35 mL/min. All samples were kept at 4 °C during the analysis. The injection volume was 2 μL. The MS was performed on an AB TripleTOF 6600 plus system, with electrospray ionization using both positive and negative ion modes. The full mass scan range was set at 100–1000 mass to charge ratio (m/z).

#### 2.4.3. Bioinformatics Data Processing

The raw data obtained from LC-MS were analyzed using Progenesis QI v2.3 (Nonlinear Dynamics, Newcastle, UK) with main parameters of 5 ppm/10 ppm precursor tolerance, 10 ppm/20 ppm product tolerance, and 5% product ion threshold. The compounds were identified based on the RT-m/z pairs. The supervised orthogonal partial least squares discriminant analysis (OPLS-DA) analysis was employed to distinguish the metabolic profiles of the NOR and REG groups, and a volcano plot was employed to visualize the alterations of the metabolite concentrations.

#### 2.4.4. Identification of the Differentially Expressed Metabolites

The metabolites were identified using Progenesis QI v2.3, according to the Human Metabolome database (http://www.hmdb.ca/, assessed on 20 June 2007), Lipid Maps database (V2.3, http://www.lipidmaps.org/, assessed 15 July on 2003), the Metlin database, and the self-built database of Shanghai Lu-Ming Biotech Co. Ltd. (Shanghai, China). To identify the DEMs in the NOR and REG groups, the thresholds were set based on the variable importance in projection (VIP) >1.0 and *p* < 0.05 from the paired Student’s *t*-test. Moreover, the DEMs were further searched using online databases, including the Aging Atlas (https://ngdc.cncb.ac.cn/aging/metabolomics, assessed on 29 October 2020) and Regeneration Roadmap (https://ngdc.cncb.ac.cn/regeneration/metabolomics, assessed on 30 September 2021).

#### 2.4.5. Pathway Enrichment Analysis

The pathway enrichment analysis for the DEMs was performed using the Kyoto Encyclopedia of Genes and Genomes database (KEGG, https://www.kegg.jp/kegg/pathway.html, assessed 22 March on 1995) through matching IDs (the KEGG ID of the corresponding DEM). A pathway with a *p*-value < 0.05 was considered a significant one.

### 2.5. Cell Culture and Differentiation Induction

#### 2.5.1. Bone Marrow-Derived Stem Cell Culture

Passage (P) 2 of human BMSCs was purchased from Haixing Biosciences (BMHX-C106, Suzhou, China). The BMSCs were cultured in the Human BMSCs Growth Medium (HyCyte, BMHX-G101, Haixing Biosciences) supplemented with 1% penicillin/streptomycin, 1% glutamine, and 10% fetal bovine serum under the condition of 5% CO_2_ in the air at 37 °C.

#### 2.5.2. Chondrogenic Differentiation

The chondrogenic differentiation in monolayer culture was induced according to the previous protocol with minor modifications [23]. Briefly, P4 BMSCs were cultured using a Human BMSCs Chondrogenic Differentiation Kit (BMHX-D203R, Haixing Biosciences) for three weeks. The differentiated chondrocytes were maintained in the chondrocyte growth medium, i.e., the DMEM/F12 medium with 10% FBS and 1% penicillin/streptomycin.

### 2.6. Stimuli and Chemicals Administrated

The oxidative stress and DNA damage to BMSCs were created by employing hydrogen peroxide (H_2_O_2_, 3%, LIRCON, Dezhou, China) and mitomycin C (MC, GC12353, GLPBIO, Montclair, CA, USA), respectively [24,25]. The dosages of H_2_O_2_ (250 μM) and MC (1 μM) were selected based on a previous study [24] and our pilot experiments (Supplementary Figure S1). The effects of UDCA (HY-13771, MCE, Monmouth Junction, NJ, USA) and DPA (GC31637, GLPBIO) on the vitality of BMSCs were examined under both control and challenge states. Various concentrations of the biomolecules (0, 5, 10, 50, and 100 µM) were added to the culture medium to determine the DEM’s contribution to the vitality of BMSCs, while the biomolecules were added with the stimulus to determine the DEM’s role under the H_2_O_2_ and MC challenged conditions. Thereafter, an optional dosage, 50 μM for both UDCA and DPA, was used in this study for identifying the effects of small molecules on chondrogenic differentiation and chondrocyte functionality.

### 2.7. Cell Vitality

The cell vitality was detected using the Cell Counting Kit-8 (CCK-8, C6005, NCM Biotech, Suzhou, China) after 24 h of incubation with and without small molecules. The optical density was read at 450 nm using a multimode plate reader (PerkinElmer, Waltham, MA, USA).

The data of the CCK-8 assay were presented as mean ± standard error (SEM). Statistical analyses and graphics were conducted using GraphPad Prism version 7.0 (San Diego, CA, USA). The effects of the small molecules under control and challenge conditions were revealed by a one-way ANOVA analysis. The Dunnett test was used to partition differences among the dosages. A value of *p* < 0.05 was considered to be statistically significant.

### 2.8. Immunofluorescence

The cells were grown on glass coverslips (ØO = 14 mm). The immunofluorescence was determined according to the method previously described with some modifications [26]. Briefly, the cells were incubated with anti-ACAN (1:500, bs-1223R, Boiss, Beijing, China) overnight at 4 °C after fixation and permeabilization. The goat anti-rabbit 594 secondary antibody (1:500, bs-0295G-AF594, Boiss) was applied for 2 h at RT. The cells were counterstained with phalloidin (C1033, Beyotime, Shanghai, China) to determine the cytoskeletal arrangement [27], and the nuclei were identified after staining with DAPI (S2110, Solarbio) for 30 min at RT. The images were taken under a Leica DM 6000B microscope (Leica, Herlev, Denmark) and were representatives of three repeats.

### 2.9. Real-Time PCR

Total RNA was extracted using NcmZol Reagent (M5100, NCM Biotech). The concentration and quality of RNA were measured using a spectrophotometer (Nanodrop 2000c, Thermo Fisher, Waltham, MA, USA). Reverse transcription was conducted using the Reverse Transcription Reagent Pack (Applied Biosystems, Thermo Fisher). Real-time PCR analysis was carried out using Quantstudio 5 (Applied Biosystems) with the SYBR Green Master Mix (Applied Vazyme, Nanjing, China) and gene-specific primers (Table 1) of the key transcriptional factor for chondrogenic differentiation (SRY-related high mobility group-box gene 9, SOX9) and the chondrogenic indicators (ACAN and COL2A1) [21,28]. The Ct-value of β-actin mRNA was used as the internal control. The relative mRNA expression levels were analyzed using the 2−ΔΔCt method [29]. The unpaired Students’ *t*-test was applied to determine the difference between DPA or UDCA treatment with the CON group. A value of *p* < 0.05 was considered to be statistically significant.

## 3. Results

### 3.1. The Histomorphologic Changes in the Regenerated Cartilage

Compared to the NOR cartilage, the REG cartilage lost its transparent color and smooth and glistening appearance, replaced with a rough surface with fissures (Figure 1B). The REG cartilage also lost the regularity seen in the H&E-stained NOR cartilage, i.e., a highly organized structure composed of four zones (Figure 1C,D). Specifically, the thickness of the REG cartilage layer at the defect site was lower than that of the NOR group, while the chondrocytes in the REG group were more compactly organized than those in the NOR group (Figure 1C–F). Furthermore, the shape of the chondrocytes in the NOR group was plumper and more round (Figure 1C,E) than that of the chondrocytes in the REG group (Figure 1D,F). Moreover, the reddish-stained ACAN content in the REG cartilage (Figure 1E) was lower than that in the NOR cartilage (Figure 1F).

### 3.2. The Metabolomic Changes in the Regenerated Cartilage

**Figure 2:**
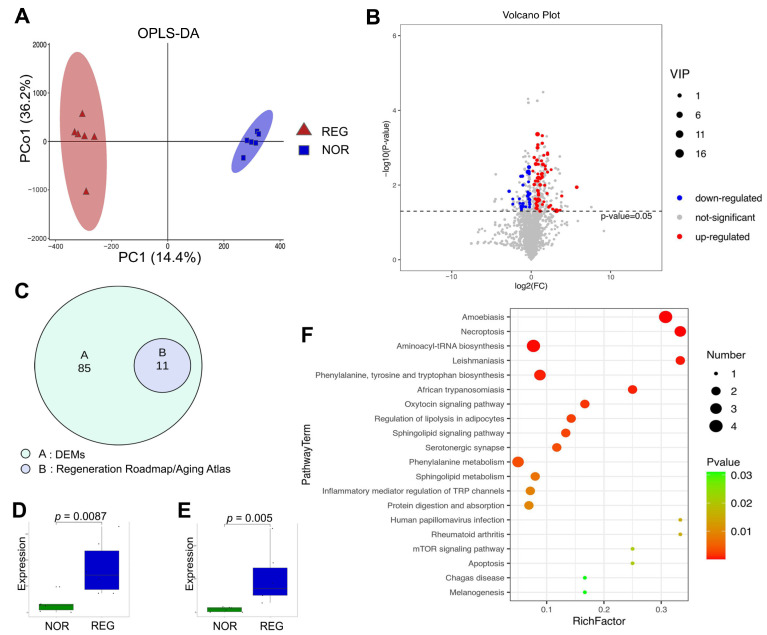
The global metabolomic profiles of the normal and regenerated cartilages. (**A**) The supervised orthogonal partial least squares discriminant analysis (OPLS-DA) model reveals the metabolic differences between the NOR and REG groups. (**B**) The volcano plot indicates the upregulated differential metabolites (DEMs) in the REG group (red), the downregulated DEMs (blue), and non-significantly altered metabolites (gray). (**C**) Venn diagram shows the DEMs matched with the records in the Regeneration Roadmap and Aging Atlas databases. (**D**,**E**) The different expression levels of docosapentaenoic acid 22n-3 (DPA, (**D**)) and ursodeoxycholic acid (UDCA, (**E**)) between the NOR and REG groups are revealed. (**F**) The bubble diagram shows the top 20 differential metabolic pathways enriched by the DEMs. *X*-axis represents rich factors, and *Y*-axis represents pathway terms. The numbers of the involved metabolites and *p*-value are listed on the right side. The detailed information is presented in Supplementary Tables S1 and S2.

The OPLS-DA plot revealed the differences in metabolomic activities between the NOR and REG groups (Figure 2A). A total of 96 DEMs were identified (Table 2), with 31 decreased and 65 increased metabolites induced by MF (Figure 2B). Of these, 11 DEMs were matched with the records in the Regeneration Roadmap and Age Atlas (Figure 2C, Supplementary Table S1). Moreover, the DEMs can be divided into six categories, including lipids and lipid-like molecules (47), organic oxygen compounds (16), organic acids and derivatives (12), heterocyclic compounds (15), organosulfur compounds (2), and hydrocarbons (4). Notably, the lipids and lipid-like molecules were accountable for half (48.96%) of the entire metabolic alterations in cartilage regeneration. Of the lipids and lipid-like molecules, the majority were increased in the REG cartilages (85.11%), including DPA (Figure 2D, *p* = 0.0087) and UDCA (Figure 2E, *p* = 0.005). The organic oxygen compounds, accounting for 16.67% of the DEMs, were mainly second messengers and oligosaccharides. Among the organic oxygen compounds, most of the glycolytic substances were decreased. The heterocyclic compounds, organosulfur compounds, and others accounted for the rest (21.88%) of the DEMs.

The enrichment analysis yielded 70 pathways related to cartilage regeneration (Supplementary Table S2), with the top 20 listed in the bubble diagram (Figure 2F), which revealed the key information associated with the biochemical-niche control for chondrogenesis, such as the rapamycin (mTOR) signaling pathway. Moreover, the detailed information on the pathways with hits ≥ 2 and *p*-value < 0.05 is presented in Table 3.

### 3.3. Differentially Expressed Metabolites Involved in Promoting the Vitality and Adaptation of BMSCs

**Figure 3:**
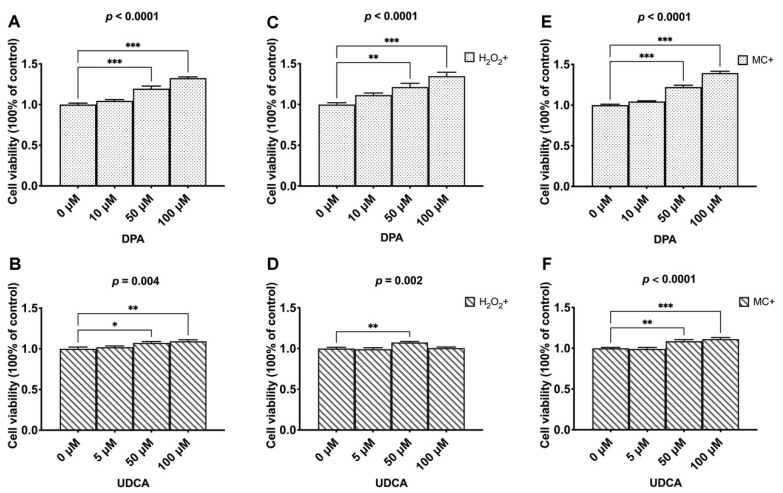
The effects of DAP and UDCA on the vitality and adaptation of the BMSCs. (**A**,**B**). The dosage effects of DPA and UDCA on the vitality of BMSCs. (**C**,**D**). The dosage effects of DPA and UDCA on the vitality of BMSCs in response to 250 μM hydrogen peroxide (H_2_O_2_)-caused oxidative stress damage. (**E**,**F**). The dosage effects of DPA and UDCA on the vitality of BMSCs in response to 1 μM mitomycin C (MC)-caused DNA damage. The data are presented as mean ± SEM (n = 7). * *p* < 0.05; ** *p* < 0.01; *** *p* < 0.0001.

The facilitating effect of both DPA (*p* < 0.0001, Figure 3A) and UDCA (*p* = 0.004, Figure 3B) on the vitality of BMSCs was revealed. Specifically, 50 and 100 μM of both DPA and UDCA significantly increased the optical densities compared to the control group (0 μM). Under oxidative challenge, adding DPA and UDCA rescued the damage effect of 250 μM H_2_O_2_ (*p* < 0.0001, Figure 3C; *p* = 0.002, Figure 3D). Similarly, the administration of DPA and UDCA rescued the injury effect of DNA damage caused by 1 μM MC (*p* < 0.0001, Figure 3E; *p* < 0.0001, Figure 3F).

### 3.4. Differentially Expressed Metabolites Involved in Promoting Chondrogenic Differentiation

**Figure 4:**
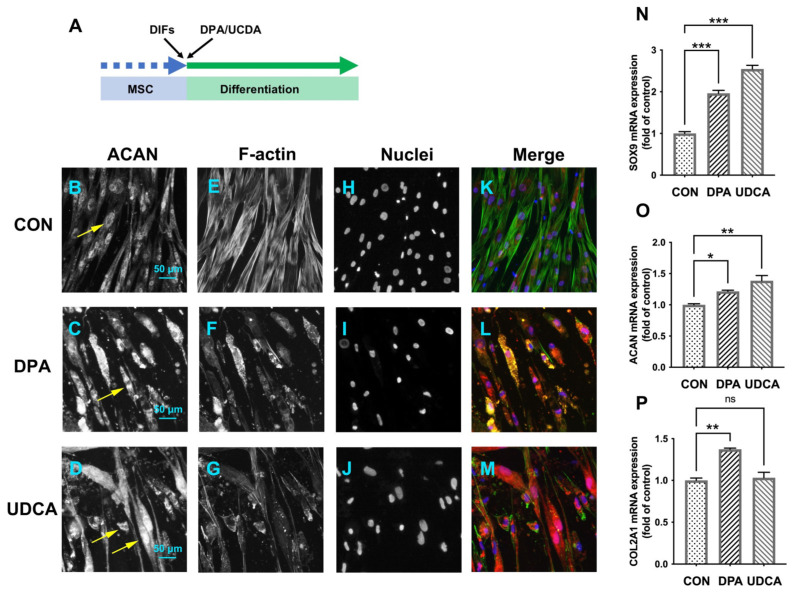
The effects of DPA and UDCA on chondrogenic differentiation. DPA and UDCA were separately administrated during the entire process of chondrogenic differentiation as indicated in (**A**). The distribution of proteoglycan aggrecan (ACAN, red) is revealed with immunofluorescence; F-actin (green) and nuclei (blue) are counterstained with phalloidin and DAPI, respectively. The immuno-positive signal of ACAN (arrows) is weak and restricted within the nucleus in the control (CON) group (**B**,**H**) and is strong and located in the cytoplasm and nucleus in both the DPA group (**C**,**I**) and UDCA group (**D**,**J**). F-actin is rearranged during the chondrogenic differentiation; i.e., it displays a sharp image after phalloidin staining in the CON group (**E**) and fades in both the DPA (**F**) and UDCA (**G**) groups. (**K**,**L**,**M**) show the merge images. The scale bar represents 50 μm. The mRNA expression levels of the SRY-related high mobility group-box gene 9 (SOX9, (**N**)), ACAN (**O**), and type II collagen (COL2A1, (**P**)) in the CON, DPA, and UDCA groups are quantified. The data are presented as mean ± SEM (n = 3). ns indicates no significance (*p* > 0.05); * *p* < 0.05, ** *p* < 0.01, *** *p* < 0.001.

After induced chondrogenic differentiation (Figure 4A), compared to the CON group (Figure 4B), the intensity of ACAN was increased in both the DPA (Figure 4C) and UDCA (Figure 4D) groups. The phalloidin staining was of high intensity, with the microfilaments easily distinguishable in the CON group (Figure 4E), while the staining in the DPA (Figure 4F) and UDCA (Figure 4G) groups was almost faded; in particular, the microfilaments in the UDCA group mostly disappeared (Figure 4G). Moreover, the ACAN signal was co-located with the nucleus in the CON group (Figure 4B,H,K) and exhibited cytoplasmic shuttling in the DPA (Figure 4C,I,L) and UDCA (Figure 4D,J,M) groups. Furthermore, the quantitative analysis indicated that the administration of DPA during chondrogenic differentiation significantly elevated the mRNA expression of SOX9 (Figure 4N), ACAN (Figure 4O), and COL2A1 (Figure 4P) compared to the CON group, while UDCA significantly increased the SOX9 (Figure 4N) and ACAN (Figure 4O) mRNA expression levels.

### 3.5. Differentially Expressed Metabolites Involved in Promoting Chondrocyte Functionality

**Figure 5:**
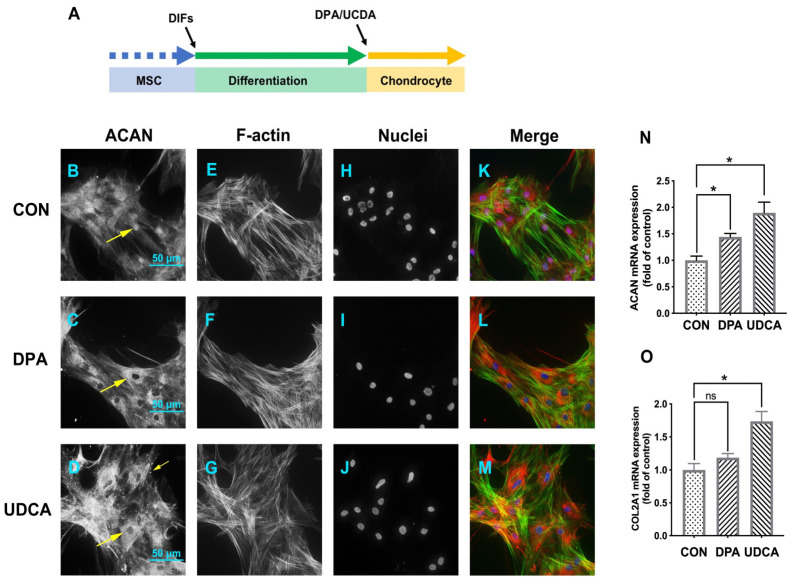
The effects of DPA and UDCA on chondrocyte functionality. DPA or UDCA was added to the chondrocytes for five days before sampling (**A**). The distribution of ACAN (red); the distribution of F-actin (green) and nuclei (blue), counterstained with phalloidin and DAPI, respectively. The immuno-positive signal of ACAN (arrows) is relatively weak and located in the nucleus and cytoplasm in the CON group (**B**,**H**,**K**), while it becomes strong, moves to the cytoplasm, and exhibits external secretion after DPA (**C**,**I**,**L**) or UDCA (**D**,**J**,**M**) administration. (**E**,**F**,**G**) indicate the shape of chondrocyte. The scale bar represents 50 μm. The mRNA expression levels of the ACAN (**N**) and COL2A1 (**O**) in the CON, DPA, and UDCA groups are quantified. The data are presented as mean ± SEM (n = 3). ns indicates no significance (*p* > 0.05); * *p* < 0.05.

The ACAN signal was detected in the nucleus and cytoplasm in the CON group (Figure 5B,H,K), while it was restricted in the cytoplasm and exhibited exocytosis (arrows) in both the DPA (Figure 5C,I,L) and UDCA (Figure 5D,J,M) groups. In addition, the chondrocytes in the CON group (Figure 5E) tended to have a fibroblast-like appearance, while those in the DPA (Figure 5F) and UDCA (Figure 5G) groups were more likely to exhibit a polygon shape. Furthermore, the quantitative analysis indicated that the ACAN mRNA expression in the chondrocytes was increased in both DPA and UDCA groups (Figure 5N), while the mRNA expression levels of COL2A1 (Figure 5O) were increased in the UDCA group only.

## 4. Discussion

Currently, the common osteoarthritis therapy with nonsteroidal anti-inflammatory drugs or chondroprotective hyaluronic acid (HA) is ineffective for cartilage regeneration [30]. The pharmaceutical industry is calling for novel biomolecules covering both safety and effectiveness as anti-aging supplements or drug candidates for joint health. The metabolomics modules in the Aging Atlas and Regeneration Roadmap offer the molecular substances potentially related to the degenerative and regenerative events in the blastema of axolotls, deer antler stem cells, etc. However, orthopedic metabolic cues are lacking. In this study, the minority of identified DEMs (11/96) were matched with the records in the Regeneration Roadmap and Aging Atlas, which stresses the uniqueness and significance of the cartilage regeneration-specific metabolites. This study firstly reveals the biomolecules for cartilage regeneration, which serve each step during MF, i.e., the vitality and adaptation of BMSCs as well as chondrogenic differentiation and chondrocyte functionality.

The vitality of the activated BMSCs may be the main restriction for cartilage regeneration, especially in old people with an inadequate BMSC pool and senescent BMSCs [5]. The vitality of BMSCs is altered by the biophysical perturbation during their migration, e.g., switched from the hypoxic bone marrow niche to the oxygen-exposed defect site in MF [31]. Oxidative metabolism, e.g., the oxidative stress and the subsequent DNA damage in the defect site, has been revealed by the increase in FAPγ-adenine in the heterocyclic compounds in the REG group, exhibiting the damage effects on the vitality of BMSCs [32]. Adding DPA or UDCA increased the vitality of the BMSCs under both the control condition and oxidative challenge. Moreover, the fluctuation in the cellular metabolism directly alters the microenvironment for cartilage regeneration in situ [33]. In this study, increases in phenylalanine, tyrosine, tryptophan, 2′-O-methyladenosine (an analog of adenosine), sphingosine, and SM(d18:1/24:1(15Z)) have been identified in the REG group. Acetyl-CoA is obtained from amino acids such as phenylalanine, tyrosine, and tryptophan [34], and oxidation of acetyl-CoA accounts for ATP production [34]. It has been reported that adenosine elevates the ATP supply in MSCs [35,36]. Moreover, the sphingolipid metabolism and sphingolipid signaling pathways are associated with stem cell migration and signaling transduction [37,38]. Hence, the DEMs may regulate the vitality of the activated BMSCs via mediating acetyl-CoA synthesis, ATP supply, and signaling transduction.

The foreign supply in the defect site is critical for the renewal and functionality of chondrocytes because cartilage is an avascular tissue [39]. The elevated second messengers in the REG group may reveal the boosting of signaling transduction in the differentiating and newborn chondrocytes. The activated mTOR signaling pathway is critical for lipid biosynthesis [40] and chondrogenic differentiation [41]. Blocking mTOR signaling inhibits chondrogenic differentiation [41]. Hence, the DEMs may participate in regulating chondrogenic differentiation and chondrocyte functionality, and they may potentially prevent cartilage loss in musculoskeletal diseases via mediating mTOR signaling transduction. In addition, both DPA and UDCA promoted chondrogenic differentiation by stimulating the mRNA expression of SOX9 and ACAN and facilitating cytoskeleton rearrangement during chondrogenic differentiation via regulating F-actin depolymerization. The administration of DPA and UDCA enhanced chondrocyte functionality by promoting the extracellular processing of ACAN. The HA-bound extracellular ACAN regulates HA endocytosis [42], which may determine the chondroprotective effect of HA. Compared to DPA, UDCA exhibited higher potential in facilitating chondrogenic differentiation and chondrocyte functionality potentially due to cholesterol’s facilitating role in chondrocyte differentiation [43]. The inconsistency between DPA and UDCA may be associated with the dosage effect or functioning pathway, and the related mechanism will be explored in further study.

## 5. Conclusions

Generally, the findings of this study (1) provided a list of biomolecules potentially involved in the fate control of the activated resident BMSCs in cartilage regeneration and (2) verified the functions of UDCA and DPA in promoting BMSC vitality, chondrogenic differentiation, and chondrocyte functionality. Ultimately, the small molecules may work as anti-aging supplements for joint rejuvenation. Clinical supply with the biomolecules in MF may confer a better outcome and promote the surgery to a broad base of patients, especially the elderly, by rebalancing and conquering the hostile joint environment. Additionally, combining the biomolecules with BMSC therapy may be a safe and effective strategy in stem cell-treated diseases, not limited to orthopedic disorders.

## Figures and Tables

**Table 1:** Gene-specific primers for real-time PCR.

Gene	Primer
SOX9	F: TAAGCTAAAGGCAACTCGTACC
	R: TAGAGAATATTCCTCACAGAGGACT
ACAN	F: TGAGCGGCAGCACTTTGAC
	R: TGAGTACAGGAGGCTTGAGG
COL2A1	F: TCCAGATGACCTTCCTACGC
	R: GGTATGTTTCGTGCAGCCAT
β-actin	F: CCCTGGAGAAGAGCTACGAG
	R: CGTACAGGTCTTTGCGGATG

**Table 2:** The list of DEMs identified in the NOR and REG groups.

Category	Metabolite	*m/z* ^a^	RT ^b^ (min)	Error ^c^ (ppm)	VIP ^d^	*p*-Value ^e^	FC ^f^	Trend	Formula
Lipids and lipid-like molecules (47)	(7Z,10Z,13Z,16Z)-docosatetraenoate	333.2783	13.8609	−2.0938	2.0825	0.0247	2.0327	up	C22H36O2
	Arachidonic acid	305.2472	13.0585	−1.1653	5.4988	0.0064	1.8881	up	C20H32O2
	Fludrocortisone acetate	405.2081	1.5828	2.2102	1.1184	0.0280	3.0242	up	C23H31FO6
	L-Palmitoylcarnitine	400.3419	10.9240	−0.6476	2.5445	0.0051	2.4512	up	C23H45NO4
	LysoPC(18:1(11Z))	522.3545	11.1461	−1.7806	2.8324	0.0022	1.6622	up	C26H52NO7P
	LysoPC(20:4(8Z,11Z,14Z,17Z))	544.3386	10.7015	−2.1933	4.1157	0.0028	2.7755	up	C28H50NO7P
	LysoPC(22:5(7Z,10Z,13Z,16Z,19Z))	570.3543	10.8646	−1.8896	1.1338	0.0039	5.7136	up	C30H52NO7P
	Prostaglandin E2	351.2173	8.2029	−1.5090	1.6011	0.0441	6.5508	up	C20H32O5
	SM(d18:1/24:1(15Z))	813.6829	13.1903	−1.8179	9.4552	0.0485	9.0986	up	C47H93N2O6P
	Sphingosine	282.2784	10.4050	−2.4257	1.4741	0.0028	1.8594	up	C18H37NO2
	DPA	331.2630	13.2346	−0.4486	1.2545	0.0349	5.5002	up	C22H34O2
	Oleamide	304.2607	13.1326	−1.4413	2.3222	0.0257	1.6991	up	C18H35NO
	Ursodeoxycholic acid	391.2857	10.7032	0.8484	1.0112	0.0472	12.4921	up	C24H40O4
	PI(O-18:0/0:0)	604.3833	14.9395	−1.7930	3.2441	0.0000	0.6783	down	C34H50O8
	LysoPE(0:0/20:4(8Z,11Z,14Z,17Z))	502.2918	10.6718	−2.0758	7.3932	0.0004	1.7392	up	C25H44NO7P
	6-[3]-ladderane-1-hexanol	280.2635	12.3763	−1.9348	6.3252	0.0251	1.7063	up	C18H30O
	PS(14:0/24:1(15Z))	840.5728	11.4733	0.3863	1.6386	0.0196	14.4043	up	C44H84NO10P
	LysoPE(22:4(7Z,10Z,13Z,16Z)/0:0)	530.3229	11.2206	−2.3436	4.2432	0.0010	1.7410	up	C27H48NO7P
	PS(14:1(9Z)/24:0)	840.5734	12.8225	1.0620	3.3834	0.0114	53.3843	up	C44H84NO10P
	Linoelaidic Acid	263.2365	13.2051	−1.6593	3.3825	0.0354	1.6614	up	C18H32O2
	Pelargonidin 3-(6″-p-coumarylglucoside)-5-(6‴-acetylglucoside)	763.1887	0.9317	0.9657	1.7822	0.0417	0.4288	down	C38H38O18
	PC(O-16:0/0:0)	482.3591	11.3248	−2.8591	2.1609	0.0008	2.6328	up	C24H52NO6P
	LysoPC(18:2(9Z,12Z))	520.3395	10.6718	−0.4349	2.1558	0.0049	2.6192	up	C26H50NO7P
	LysoPE(0:0/18:2(9Z,12Z))	476.2778	10.6394	−1.0077	1.3499	0.0031	3.4070	up	C23H44NO7P
	LysoPE(22:5(7Z,10Z,13Z,16Z,19Z)/0:0)	528.3073	10.8350	−2.1896	2.5262	0.0015	4.1787	up	C27H46NO7P
	1-O-(2R-hydroxy-hexadecyl)-sn-glycerol	355.2815	12.7332	−2.7314	2.4189	0.0170	0.8881	down	C19H40O4
	2,3,4,5,2′,3′,4′,6′-Octamethoxychalcone	895.3420	0.7166	2.9042	1.6332	0.0189	0.7754	down	C23H28O9
	15-hydroxy-tetracosa-6,9,12,16,18-pentaenoic acid	357.2786	10.7015	−0.4361	1.5497	0.0168	4.0203	up	C24H38O3
	Xestoaminol C	230.2473	9.4959	−2.5076	1.9590	0.0078	1.4550	up	C14H31NO
	LysoPE(18:2(9Z,12Z)/0:0)	478.2919	10.6421	−1.8674	1.8550	0.0005	2.6353	up	C23H44NO7P
	1,2-(8R,9R-epoxy-17E-octadecen-4,6-diynoyl)-3-(hexadecanoyl)sn-glycerol	839.5820	11.9032	−0.0844	1.4486	0.0101	3.2667	up	C54H80O8
	1-(11Z,14Z-eicosadienoyl)-glycero-3-phosphate	507.2730	11.5926	0.2704	1.2619	0.0014	4.1891	up	C23H43O7P
	Stearoylcarnitine	428.3724	11.4139	−2.4569	1.5537	0.0045	4.1705	up	C25H49NO4
	1-O-(2R-hydroxy-tetradecyl)-sn-glycerol	327.2499	11.9180	−3.8832	1.1413	0.0050	0.8497	down	C17H36O4
	LysoPE(0:0/22:5(7Z,10Z,13Z,16Z,19Z))	528.3073	11.0426	−2.2949	1.3834	0.0059	2.6454	up	C27H46NO7P
	1-(2-methoxy-eicosanyl)-sn-glycero-3-phosphoethanolamine	508.3751	11.5454	−2.0199	1.5825	0.0022	2.4697	up	C26H56NO7P
	PC(0:0/18:1(9Z))	566.3471	11.1411	1.5304	1.5653	0.0258	2.0328	up	C26H52NO7P
	N-(3-oxo-butanoyl)-homoserine lactone	186.0753	0.9084	−4.0254	1.1941	0.0065	1.7216	up	C8H11NO4
	PS(17:0/0:0)	512.2973	11.6489	−1.9114	1.0334	0.0236	0.2002	down	C23H46NO9P
	Oleoylcarnitine	426.3569	11.0426	−2.1469	1.1955	0.0417	1.4961	up	C25H47NO4
	Linoleamide	302.2448	12.3763	−2.2846	1.0171	0.0368	1.6485	up	C18H33NO
	1-Arachidonoylglycerophosphoinositol	603.2915	10.9093	−2.2292	1.1105	0.0037	3.4729	up	C29H49O12P
	Dodecanoylcarnitine	344.2790	9.7305	−1.6266	1.3234	0.0324	0.2012	down	C19H37NO4
	1-(2-methoxy-13-methyl-tetradecanyl)-sn-glycero-3-phosphoserine	522.2814	10.7905	2.3016	1.1583	0.0050	1.7855	up	C22H46NO9P
	Europinidin	330.0742	4.4330	−1.1294	1.1275	0.0143	1.8673	up	C16H13O5+
	LysoPC(P-16:0)	480.3432	11.3099	−3.5157	1.5174	0.0234	1.8926	up	C24H50NO6P
	(E)-2-Penten-1-ol	104.1067	0.7885	−3.0443	2.9246	0.0097	1.3086	up	C5H10O
Organic oxygen compounds (16)	D-Myoinositol 4-phosphate	259.0220	0.7671	−1.6433	2.6807	0.0147	0.1456	down	C6H13O9P
	N-Acetylgalactosamine	244.0790	0.8485	−0.5407	1.0594	0.0236	1.7493	up	C8H15NO6
	D-glycero-L-galacto-Octulose	279.0470	0.7739	−2.7275	1.2166	0.0385	0.6264	down	C8H16O8
	Pelargonidin	272.0673	4.4259	−2.2420	2.8195	0.0058	0.4624	down	C15H11O5+
	Malvidin	332.0888	4.4543	1.6129	3.5305	0.0099	2.1895	up	C17H15O7+
	Cellotetraose	684.2545	0.9383	−1.7043	3.2822	0.0375	0.4180	down	C24H42O21
	N-Acetylgalactosaminyl lactose	546.2017	0.9232	−2.0868	2.2153	0.0058	0.4044	down	C20H35NO16
	Isopropyl β-D-ThiogalactoPyranoside	221.0837	2.0908	−1.9212	4.9497	0.0033	0.8273	down	C9H18O5S
	3,5-dihydroxy-4-(sulfooxy)benzoic acid	250.9848	0.8485	−3.1660	1.6067	0.0008	1.7730	up	C7H6O8S
	1,2,3,4-Tetramethoxy-5-(2-propenyl)benzene	261.1095	8.1917	−0.7636	2.3229	0.0175	0.8684	down	C13H18O4
	Vicianose	295.1024	2.0365	0.1620	1.6166	0.0289	0.8576	down	C11H20O10
	2-Deoxy-D-ribose 1,5-bisphosphate	292.9836	8.1902	1.0583	1.3599	0.0082	0.7965	down	C5H12O10P2
	Lacto-N-triaose	590.1927	0.9317	−2.5947	1.0686	0.0309	0.3734	down	C20H35NO16
	2-(5,8-Tetradecadienyl)cyclobutanone	245.2258	13.2051	−2.3519	1.1487	0.0368	1.7212	up	C18H30O
	4-Acetylzearalenone	361.1658	13.0585	3.5409	1.1157	0.0164	1.8800	up	C20H24O6
	Myrigalone H	304.1544	11.4883	0.2525	1.0970	0.0436	1.7111	up	C17H18O4
Organic acids and derivatives (12)	L-Arginine	175.1181	0.7447	−4.9509	1.1077	0.0232	1.4089	up	C6H14N4O2
	L-Phenylalanine	166.0856	3.5249	−4.1785	2.1999	0.0140	1.7636	up	C9H11NO2
	L-Tryptophan	203.0824	4.3576	−1.1300	1.1625	0.0066	1.8097	up	C11H12N2O2
	L-Tyrosine	180.0665	2.2998	−0.8408	1.9941	0.0065	1.7151	up	C9H11NO3
	7,8-diaminononanoic acid	171.1484	6.5341	−4.3633	1.8274	0.0198	0.7210	down	C9H20N2O2
	Dopaxanthin	779.2082	0.9317	3.6779	1.0294	0.0464	0.4276	down	C18H18N2O8
	6-(5-carboxy-2-hydroxy-3-methoxyphenoxy)-3,4,5-trihydroxyoxane-2-carboxylic acid	361.0779	0.9383	3.8961	1.3373	0.0374	0.5079	down	C14H16O11
	S-Glutathionyl-L-cysteine	427.0942	0.9232	−2.2858	1.7851	0.0390	4.6303	up	C13H22N4O8S2
	Cysteineglutathione disulfide	425.0798	0.9189	−2.0096	1.7524	0.0369	4.9326	up	C13H22N4O8S2
	Propanoyl phosphate	306.9978	9.7584	−3.7713	1.3013	0.0154	0.7428	down	C3H7O5P
	2-Hydroxycinnamic acid	182.0804	2.3357	−4.7335	2.6129	0.0184	1.9098	up	C9H8O3
	Hydroxymethylphosphonate	110.9848	0.8047	−3.7569	1.1112	0.0311	1.5147	up	CH5O4P
Heterocyclic Compounds (15)	Uracil	113.0342	1.2618	−3.2771	1.4253	0.0280	1.7119	up	C4H4N2O2
	Deethylatrazine	188.0701	4.3813	−0.5980	2.6772	0.0062	2.5574	up	C6H10ClN5
	dirithromycin	563.5503	13.1326	−4.2918	3.0670	0.0497	2.1749	up	C39H72
	2′-O-Methyladenosine	282.1185	3.3656	−4.2975	1.1541	0.0233	0.3554	down	C11H15N5O4
	FAPy-adenine	171.0985	14.2547	−2.7181	10.0091	0.0045	0.7720	down	C5H7N5O
	Pectachol	460.2687	10.2578	−1.3892	3.0608	0.0237	0.8238	down	C26H34O6
	1-(1,2,3,4,5-Pentahydroxypent-1-yl)-1,2,3,4-tetrahydro-beta-carboline-3-carboxylate	367.1491	3.3656	−2.4233	1.6882	0.0314	0.4717	down	C17H22N2O7
	Nocodazole	300.0449	0.8047	0.1909	1.7345	0.0100	0.4874	down	C14H11N3O3S
	Dipyridamole	527.3043	12.0652	1.5729	1.2210	0.0388	0.8693	down	C20H39N5O10
	6-Methyltetrahydropterin	204.0861	0.9084	2.7681	1.2488	0.0079	1.8114	up	C7H11N5O
	Gravacridonetriol	396.0855	0.0425	3.1419	1.5097	0.0248	0.8872	down	C19H19NO6
	Nicorandil	256.0578	0.9062	1.3308	1.0936	0.0068	2.2259	up	C8H9N3O4
	Ethosuximide M5	200.0562	2.2873	−1.8422	1.1403	0.0168	1.9016	up	C7H9NO3
	Dihydrofolic acid	424.1355	2.2873	−4.4941	1.2621	0.0016	3.0187	up	C19H21N7O6
	AFN911	550.2322	0.9232	−1.0333	1.0043	0.0310	0.5018	down	C29H33N7O2
Organosulfur compounds (2)	Ethyl isopropyl disulfide	137.0456	1.4293	1.9879	5.5947	0.0027	1.5142	up	C5H12S2
	Ethyl propyl disulfide	135.0312	1.4392	3.3806	2.2132	0.0018	1.3007	up	C5H12S2
Hydrocarbons and derivatives (4)	2-(Fluoromethoxy)-1,1,3,3,3-pentafluoro-1-propene (Compound A)	358.9943	8.0025	−1.1090	2.5442	0.0177	0.7840	down	C4H2F6O
	(+/−)-N,N-Dimethyl menthyl succinamide	186.2209	14.2250	−4.3869	2.2373	0.0327	0.8893	down	C12H24
	2-Hexylidenecyclopentanone	331.2642	13.8606	−0.0544	1.2698	0.0331	2.6671	up	C11H18O
	Aluminium dodecanoate	663.4544	14.9840	0.4274	1.1638	0.0042	1.1576	up	C36H69AlO6

^a^ mass to charge ratio of the features; ^b^ retention time of the features; ^c^ mass error is obtained by using the experimental mass minus the theoretical mass; ^d^ variable importance in projection; ^e^ *p*-value is obtained from the two-tailed Student’s *t*-test; and ^f^ fold change.

**Table 3:** The list of the potential pathways involved in cartilage regeneration.

NO.	Annotation	*p*-Value ^a^	Match Status ^b^	Rich Factor ^c^	Matching IDs	DEMs
1	Amoebiasis	0.0000	4/13	0.3077	C00062 C00219 C00584 C01074	L-Arginine, Arachidonic acid, Prostaglandin E2, N-Acetylgalactosamine
2	Necroptosis	0.0000	3/9	0.3333	C00219 C00319 C00550	Arachidonic acid, Sphingosine, SM(d18:1/24:1(15Z))
3	Aminoacyl-tRNA biosynthesis	0.0001	4/52	0.0769	C00062 C00078 C00079 C00082	L-Arginine, L-Tryptophan, L-Phenylalanine, L-Tyrosine
4	Leishmaniasis	0.0004	2/6	0.3333	C00219 C00584	Arachidonic acid, Prostaglandin E2
5	Phenylalanine, tyrosine, and tryptophan biosynthesis	0.0006	3/34	0.0882	C00078 C00079 C00082	L-Tryptophan, L-Phenylalanine, L-Tyrosine
6	African trypanosomiasis	0.0007	2/8	0.2500	C00078 C00584	L-Tryptophan, Prostaglandin E2
7	Oxytocin signaling pathway	0.0017	2/12	0.1667	C00219 C00584	Arachidonic acid, Prostaglandin E2
8	Regulation of lipolysis in adipocytes	0.0023	2/14	0.1429	C00219 C00584	Arachidonic acid, Prostaglandin E2
9	Sphingolipid signaling pathway	0.0026	2/15	0.1333	C00319 C00550	Sphingosine, SM(d18:1/24:1(15Z))
10	Serotonergic synapse	0.0034	2/17	0.1176	C00078 C00219	L-Tryptophan, Arachidonic acid
11	Phenylalanine metabolism	0.0034	3/60	0.0500	C00079 C00082 C01772	L-Phenylalanine, L-Tyrosine, 2-Hydroxycinnamic acid
12	Sphingolipid metabolism	0.0072	2/25	0.0800	C00319 C00550	Sphingosine, SM(d18:1/24:1(15Z))
13	Inflammatory mediator regulation of TRP channels	0.0090	2/28	0.0714	C00219 C00584	Arachidonic acid, Prostaglandin E2
14	Protein digestion and absorption	0.0097	2/29	0.0690	C00062 C00079	L-Arginine, L-Phenylalanine
15	Biosynthesis of unsaturated fatty acids	0.0494	2/69	0.0290	C00219 C16527	Arachidonic acid

The pathways listed in Table 3 have hits ≥ 2 and *p*-value < 0.05. ^a^ *p*-value is obtained by $1-\overset{m-1}{\underset{i=0}{\sum }}\frac{\left(\begin{array}{c} M\\ i \end{array}\right)\left(\begin{array}{c} N-M\\ n-i \end{array}\right)}{\left(\begin{array}{c} N\\ n \end{array}\right)}$, where *N* represents the number of the total metabolites, *n* represents the number of the DEMs, *M* represents the number of the total metabolites in a certain pathway, and *m* represents the number of DEMs in a certain pathway; ^b^ match status is indicated as $\frac{m}{M}$; ^c^ rich factor is identified as $\frac{m}{M}$.

## Data Availability

Data are available from the corresponding author under reasonable request.

## Supplementary Materials

The following supporting information can be downloaded at: https://www.mdpi.com/article/10.3390/cells11192951/s1, Figure S1: The dosages of the stimuli. Various concentrations of H_2_O_2_ (A) and MC (B) were added in the culture medium and co-incubated with the BMSCs for 24 h, and the cell viability was decided by CCK8 assay. The data of cell viability assay were presented as MEAN ± standard error (SEM). A one-way ANOVA analysis was employed to determine the damage effect of H_2_O_2_ and MC. The Dunnett test was used to partition differences of each stimulus-treated group with the control (0 μM); Table S1: The DEMs matched with the records in Regeneration Roadmap and Aging Atlas; Table S2: The list of the potential pathways induced by MF.

## References

[1] Roseti L., Desando G., Cavallo C., Petretta M., Grigolo B. (2019). Articular Cartilage Regeneration in Osteoarthritis. Cells.

[2] Ramasamy T.S., Yee Y.M., Khan I.M. (2021). Chondrocyte Aging: The Molecular Determinants and Therapeutic Opportunities. Front. Cell Dev. Biol..

[3] Andia I., Maffulli N. (2017). Biological Therapies in Regenerative Sports Medicine. Sports Med..

[4] Pittenger M.F., Mackay A.M., Beck S.C., Jaiswal R.K., Douglas R., Mosca J.D., Moorman M.A., Simonetti D.W., Craig S., Marshak D.R. (1999). Multilineage Potential of Adult Human Mesenchymal Stem Cells. Science.

[5] Im G.-I. (2016). Endogenous Cartilage Repair by Recruitment of Stem Cells. Tissue Eng. Part B Rev..

[6] Steadman J.R., Briggs K.K., Rodrigo J.J., Kocher M.S., Gill T.J., Rodkey W.G. (2003). Outcomes of microfracture for traumatic chondral defects of the knee: Average 11-year follow-up. Arthrosc. J. Arthrosc. Relat. Surg..

[7] Lee J.M., Kim B.-S., Lee H., Im G.-I. (2012). In Vivo Tracking of Mesechymal Stem Cells Using Fluorescent Nanoparticles in an Osteochondral Repair Model. Mol. Ther..

[8] Armiento A.R., Alini M., Stoddart M.J. (2018). Articular fibrocartilage—Why does hyaline cartilage fail to repair?. Adv. Drug Deliv. Rev..

[9] Morrison S.J., Spradling A.C. (2008). Stem Cells and Niches: Mechanisms That Promote Stem Cell Maintenance throughout Life. Cell.

[10] Oh J., Lee Y.D., Wagers A.J. (2014). Stem cell aging: Mechanisms, regulators and therapeutic opportunities. Nat. Med..

[11] Ward O.P., Singh A. (2005). Omega-3/6 fatty acids: Alternative sources of production. Process Biochem..

[12] Lee M.Y., Ryu J.M., Lee S.H., Park J.H., Han H.J. (2010). Lipid rafts play an important role for maintenance of embryonic stem cell self-renewal. J. Lipid Res..

[13] Wu K.K. (2021). Control of Mesenchymal Stromal Cell Senescence by Tryptophan Metabolites. Int. J. Mol. Sci..

[14] Denu R.A., Hematti P. (2016). Effects of Oxidative Stress on Mesenchymal Stem Cell Biology. Oxid. Med. Cell. Longev..

[15] Ito K., Hirao A., Arai F., Matsuoka S., Takubo K., Hamaguchi I., Nomiyama K., Hosokawa K., Sakurada K., Nakagata N. (2004). Regulation of oxidative stress by ATM is required for self-renewal of haematopoietic stem cells. Nature.

[16] Yu J., Shi J., Zhang Y., Zhang Y., Huang Y., Chen Z., Yang J. (2018). The replicative senescent mesenchymal stem/stromal cells defect in DNA damage response and anti-oxidative capacity. Int. J. Med. Sci..

[17] Lepetsos P., Papavassiliou A.G. (2016). ROS/oxidative stress signaling in osteoarthritis. Biochim. Biophys. Acta (BBA) Mol. Basis Dis..

[18] Zainal Z., Longman A., Hurst S., Duggan K., Caterson B., Hughes C., Harwood J. (2009). Relative efficacies of omega-3 polyunsaturated fatty acids in reducing expression of key proteins in a model system for studying osteoarthritis. Osteoarthr. Cartil..

[19] Chen H., Chevrier A., Hoemann C., Sun J., Lascau-Coman V., Buschmann M. (2013). Bone marrow stimulation induces greater chondrogenesis in trochlear vs condylar cartilage defects in skeletally mature rabbits. Osteoarthr. Cartil..

[20] Zu Y., Mu Y., Li Q., Zhang S.-T., Yan H.-J. (2019). Icariin alleviates osteoarthritis by inhibiting NLRP3-mediated pyroptosis. J. Orthop. Surg. Res..

[21] Thorp H., Kim K., Kondo M., Grainger D.W., Okano T. (2020). Fabrication of hyaline-like cartilage constructs using mesenchymal stem cell sheets. Sci. Rep..

[22] Shao Q., Cheng J., Li Y., Ni G. (2020). Liquid Chromatography-Mass Spectrometry-Based Plasma Metabolomics Study of the Effects of Moxibustion with Seed-Sized Moxa Cone on Hyperlipidemia. Evid. Based Complement. Altern. Med..

[23] Ruhl T., Beier J.P. (2019). Quantification of chondrogenic differentiation in monolayer cultures of mesenchymal stromal cells. Anal. Biochem..

[24] Cremers N.A.J., Lundvig D.M.S., Van Dalen S.C.M., Schelbergen R.F., Van Lent P.L.E.M., Szarek W.A., Regan R.F., Carels C.E., Wagener F.A.D.T.G. (2014). Curcumin-Induced Heme Oxygenase-1 Expression Prevents H2O2-Induced Cell Death in Wild Type and Heme Oxygenase-2 Knockout Adipose-Derived Mesenchymal Stem Cells. Int. J. Mol. Sci..

[25] Cheng S.-Y., Seo J., Huang B.T., Napolitano T., Champeil E. (2016). Mitomycin C and decarbamoyl mitomycin C induce p53-independent p21WAF1/CIP1 activation. Int. J. Oncol..

[26] Huang X., Kuang S., Applegate T.J., Lin T.-L., Cheng H.-W. (2021). Prenatal Serotonin Fluctuation Affects Serotoninergic Development and Related Neural Circuits in Chicken Embryos. Neuroscience.

[27] Takano I., Takeshita N., Yoshida M., Seki D., Oyanagi T., Kimura S., Jiang W., Sasaki K., Sogi C., Kawatsu M. (2020). Ten-m/Odz3 regulates migration and differentiation of chondrogenic ATDC5 cells via RhoA-mediated actin reorganization. J. Cell. Physiol..

[28] Kou I., Ikegawa S. (2004). SOX9-dependent and -independent Transcriptional Regulation of Human Cartilage Link Protein. J. Biol. Chem..

[29] Livak K.J., Schmittgen T.D. (2001). Analysis of relative gene expression data using real-time quantitative PCR and the 2^−ΔΔCT^ Method. Methods.

[30] Jerosch J. (2011). Effects of Glucosamine and Chondroitin Sulfate on Cartilage Metabolism in OA: Outlook on Other Nutrient Partners Especially Omega-3 Fatty Acids. Int. J. Rheumatol..

[31] Parate D., Kadir N.D., Celik C., Lee E.H., Hui J.H.P., Franco-Obregón A., Yang Z. (2020). Pulsed electromagnetic fields potentiate the paracrine function of mesenchymal stem cells for cartilage regeneration. Stem Cell Res. Ther..

[32] Cadet J., Wagner J.R. (2013). DNA Base Damage by Reactive Oxygen Species, Oxidizing Agents, and UV Radiation. Cold Spring Harb. Perspect. Biol..

[33] Shah S., Esdaille C.J., Bhattacharjee M., Kan H.-M., Laurencin C.T. (2022). The synthetic artificial stem cell (SASC): Shifting the paradigm of cell therapy in regenerative engineering. Proc. Natl. Acad. Sci. USA.

[34] Akram M. (2014). Citric acid cycle and role of its intermediates in metabolism. Cell Biochem. Biophys..

[35] Jeske S.S., Theodoraki M.N., Boelke E., Laban S., Brunner C., Rotter N., Jackson E.K., Hoffmann T.K., Schuler P.J. (2020). Adenosine production in mesenchymal stromal cells in relation to their developmental status. HNO.

[36] Diamante L., Martello G. (2022). Metabolic regulation in pluripotent stem cells. Curr. Opin. Genet. Dev..

[37] Spiegel S., Milstien S. (2007). Functions of the Multifaceted Family of Sphingosine Kinases and Some Close Relatives. J. Biol. Chem..

[38] Ullah I., Subbarao R.B., Rho G.J. (2015). Human mesenchymal stem cells—Current trends and future prospective. Biosci. Rep..

[39] Villalvilla A., Gómez R., Largo R., Herrero-Beaumont G. (2013). Lipid Transport and Metabolism in Healthy and Osteoarthritic Cartilage. Int. J. Mol. Sci..

[40] Rivas D.A., Yaspelkis B.B., Hawley J.A., Lessard S.J. (2009). Lipid-induced mTOR activation in rat skeletal muscle reversed by exercise and 5′-aminoimidazole-4-carboxamide-1-β-d-ribofuranoside. J. Endocrinol..

[41] Li H., Jin Y., Zhao Y., Li W., He Z., Zhang Q., Huang H., Lin J., Chen Y., Xing D. (2021). Targeted cell therapy for partial-thickness cartilage defects using membrane modified mesenchymal stem cells by transglutaminase 2. Biomaterials.

[42] Danielson B.T., Knudson C.B., Knudson W. (2015). Extracellular Processing of the Cartilage Proteoglycan Aggregate and Its Effect on CD44-mediated Internalization of Hyaluronan. J. Biol. Chem..

[43] Gentili C., Tutolo G., Pianezzi A., Cancedda R., Cancedda F.D. (2005). Cholesterol secretion and homeostasis in chondrocytes: A liver X receptor and retinoid X receptor heterodimer mediates apolipoprotein A1 expression. Matrix Biol..

