# Supplementary material for: Cartilaginous Metabolomics Reveals the Biochemical-Niche Fate Control of Bone Marrow-Derived Stem Cells
Source: Cells. 2022 Sep 21;11(19):2951. doi: 10.3390/cells11192951 (PMC9562901; doi:10.3390/cells11192951)
Supplement: Supplementary file 1 [file cells-11-02951-s001.zip › Supplementary Figure S1.pdf]

Supplementary Figure

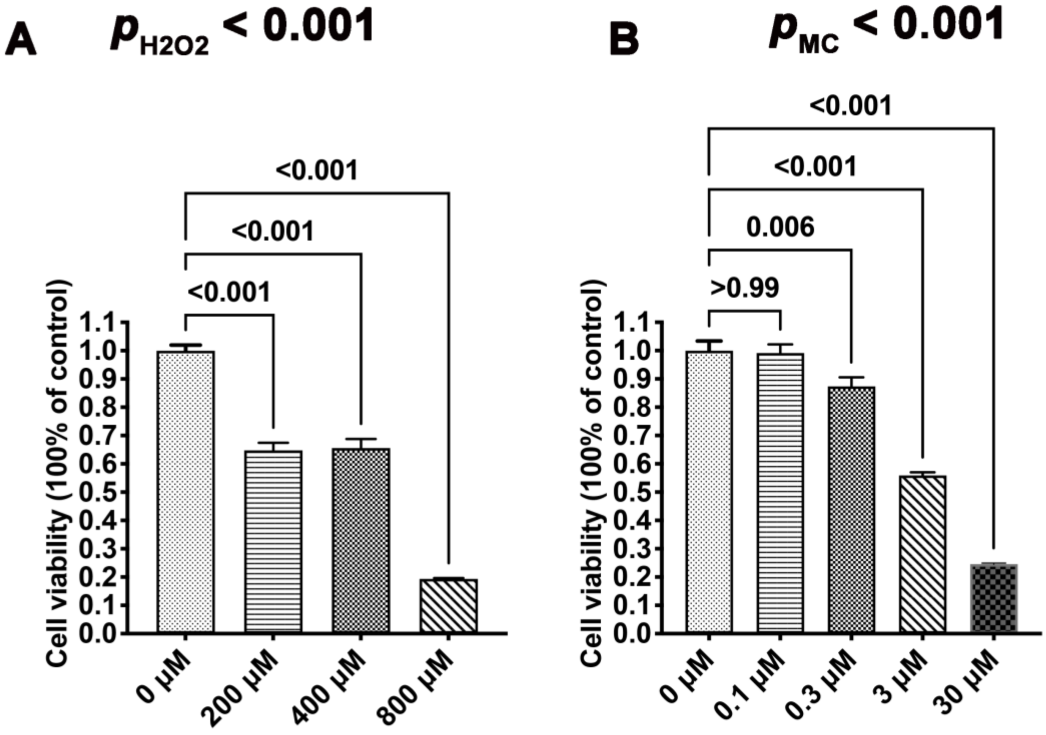

Supplementary Figure S1. The dosages of the stimuli. Various concentrations of H_2O_2 (A) and MC (B) were added in the culture medium and co-incubated with the BMSCs for 24 h, and the cell viability was decided by CCK8 assay. The data of cell viability assay were presented as MEAN ± standard error (SEM). A one-way ANOVA analysis was employed to determine the damage effect of H_2O_2 and MC. The Dunnett test was used to partition differences of each stimulus-treated group with the control (0 μM).
